# Attractiveness Compensates for Low Status Background in the Prediction of Educational Attainment

**DOI:** 10.1371/journal.pone.0155313

**Published:** 2016-06-01

**Authors:** Shawn Bauldry, Michael J. Shanahan, Rosemary Russo, Brent W. Roberts, Rodica Damian

**Affiliations:** 1 Department of Sociology, University of Alabama-Birmingham, Birmingham, Alabama, United States of America; 2 Department of Sociology, University of North Carolina at Chapel Hill, Chapel Hill, North Carolina, United States of America; 3 Department of Psychology, University of Illinois at Urbana-Champaign, Urbana-Champaign, Illinois, United States of America; 4 Department of Psychology, University of Houston, Houston, Texas, United States of America; Stony Brook University, Graduate Program in Public Health, UNITED STATES

## Abstract

**Background:**

People who are perceived as good looking or as having a pleasant personality enjoy many advantages, including higher educational attainment. This study examines (1) whether associations between physical/personality attractiveness and educational attainment vary by parental socioeconomic resources and (2) whether parental socioeconomic resources predict these forms of attractiveness. Based on the theory of resource substitution with structural amplification, we hypothesized that both types of attractiveness would have a stronger association with educational attainment for people from disadvantaged backgrounds (resource substitution), but also that people from disadvantaged backgrounds would be less likely to be perceived as attractive (amplification).

**Methods:**

This study draws on data from the National Longitudinal Study of Adolescent to Adult Health—including repeated interviewer ratings of respondents’ attractiveness—and trait-state structural equation models to examine the moderation (substitution) and mediation (amplification) of physical and personality attractiveness in the link between parental socioeconomic resources and educational attainment.

**Results:**

Both perceived personality and physical attractiveness have stronger associations with educational attainment for people from families with lower levels of parental education (substitution). Further, parental education and income are associated with both dimensions of perceived attractiveness, and personality attractiveness is positively associated with educational attainment (amplification). Results do not differ by sex and race/ethnicity. Further, associations between perceived attractiveness and educational attainment remain after accounting for unmeasured family-level confounders using a sibling fixed-effects model.

**Conclusions:**

Perceived attractiveness, particularly personality attractiveness, is a more important psychosocial resource for educational attainment for people from disadvantaged backgrounds than for people from advantaged backgrounds. People from disadvantaged backgrounds, however, are less likely to be perceived as attractive than people from advantaged backgrounds.

## Introduction

People who are perceived as good looking or as having a pleasant personality enjoy a host of social advantages. Physical attractiveness has a well-established link with earnings and other desirable job-related outcomes [1–9]. Indeed, one study suggests that the return on attractiveness is comparable in magnitude to the return on ability [2]. Studies have also identified effects of perceived physical attractiveness on educational outcomes, including teacher’s perceptions of academic ability, grade point average (GPA), and educational attainment [10–14].

Associations between perceived personality attractiveness and attainments have received far less attention than perceived physical attractiveness, and studies report mixed results [10,15]. Although not measuring an attractive personality per se, numerous studies have examined the effects of different dimensions of personality on educational and labor market outcomes [16–21]. Some dimensions of personality—especially, in the American context, agreeableness and extraversion—likely foster perceptions of an attractive personality. Research has identified associations of agreeableness with GPA and educational attainment [20,22] and of extraversion with job performance and work conditions [20,23]. Of the five dimensions of personality recognized by the NEO Personality Inventory, conscientiousness is most consistently related to indicators of attainment, GPA, earnings, and occupational prestige [22,24–27].

This paper extends this research in four respects. First, the association between attractiveness and attainments may vary by parental status such that attractiveness is a resource that compensates for low SES background. That is, the association between attractiveness and attained education may be stronger for children from low socioeconomic backgrounds. Second, attractiveness is one potential mechanism by which attainments reproduce across the generations. This mediational hypothesis (i.e., parental status → attractiveness → attained education) stems from the idea that higher status parents have the resources to foster attractiveness in their children, which in turn evokes more positive assessments from other people (e.g., teachers and employers). Third, past studies typically focus on measures of attractiveness at one point in time. This study, in contrast, extracts the stable trait component of attractiveness from three measures spanning the time period from adolescence to young adulthood and thus addresses measurement error that arises from a reliance on measuring attractiveness at a single time point. Finally, most past studies focus on one dimension of attractiveness—either physical or personality—at a time despite the fact that they are likely correlated (but see [10] for an exception). This study simultaneously assesses both physical and personality attractiveness in the same models.

### Attractiveness As a Substituted Resource

Drawing on Ross and Mirowsky’s work on resource substitution and structural amplification [28–31], we hypothesize that perceived attractiveness, both physical and personality, may have a stronger association with educational attainment at lower levels of parent socioeconomic resources. The resource substitution hypothesis states that a given psychosocial resource will be more beneficial for people who have fewer alternative psychosocial resources. For instance, education influences health moreso among people with less (versus highly) educated parents [31].

In the present context, perceived attractiveness represents a psychosocial resource that fosters educational attainment and may function as an alternate resource when family socioeconomic resources are lacking. Youth whose behaviors comport to middle-class expectations are known to fare better in classrooms, even with adjustments for intellectual ability [32]. Such youth are less disruptive and they constructively contribute to the social dynamics of the classroom, which teachers reward both explicitly and implicitly. For example, youth who are competent, goal- driven, and easy to get along with may appear a “better investment” of a teacher’s time than youth who are impulsive and behaviorally challenging [33]. Indeed, attractive youth from low status households may be more likely to elicit support from teachers, and these relationships are positively associated with educational continuation [34]. Consistent with these mechanisms, both perceived physical and personality attractiveness may, in part, compensate for some of the disadvantages associated with a poor socioeconomic background.

### Attractiveness and Intergenerational Status

Parent socioeconomic resources may enhance perceptions of their children’s attractiveness via several mechanisms. First, economic resources could directly influence perceptions of attractiveness by enabling children to purchase fashionable clothing and other accoutrements of wealth and status. Second, parent socioeconomic resources tend to be associated with adopting healthier lifestyles that in turn may increase perceptions of attractiveness. Parental education is associated with knowledge about health and diet, and higher income allows people to buy healthier, fresh foods and to enroll their children in sports camps, gyms, and other activities. In turn, children of higher status parents have lower body mass indices and such people tend to be perceived as more attractive [35,36]. Third, parent socioeconomic resources are known to be associated with various dimensions of personality that are likely to be factors in ratings of perceived attractiveness [20,37,38]. For instance, one study found that higher levels of parental education are associated with higher ratings of agreeableness, extraversion, and imagination and lower ratings of neuroticism among their adult children [20].

If perceived attractiveness has a greater effect on educational attainment for people with less socioeconomic resources (i.e., moderation) and perceived attractiveness is a mechanism through which parent socioeconomic resources shape their children’s educational attainment (i.e., mediation), then a pattern emerges that Ross and Mirowsky refer to as “structural amplification” [20,29,39]. In the context of this study, structural amplification refers to the possibility that those youth most likely to benefit from perceived attractiveness will be least likely to be perceived as attractive.

## Data and Methods

Data for this analysis come from the National Longitudinal Study of Adolescent to Adult Health (Add Health). Wave I of Add Health is a nationally representative sample of adolescents who were enrolled in middle school or high school in 1994. The sample was obtained by first randomly selecting 80 high schools from a list of all high schools in the United States stratified to ensure adequate representation of different regions, types of schools, ethnic compositions of schools, and study body sizes. The 80 high schools were then paired with 65 middles schools that fed into their student body. The combined 145 middle and high schools hosted an in-school survey that yielded 90,118 student respondents in grades 7 through 12 in 1994. Approximately 200 students from each school were randomly selected for in-depth in-home interviews, which resulted in a sample of 20,745 adolescents at Wave I. The in-home component also included an interview with a parent or caretaker (typically the mother or female head of the household if available). The parent/caretaker interviews included information about parent socioeconomic resources that is used in the following analyses.

A second wave of data was collected roughly one year after the first wave and included N = 14,738 respondents. A third wave of data was collected in 2001–02, roughly seven years after the first wave of data. The third wave included 15,197 respondents between the ages of 18 and 28. Finally, a fourth wave of data was collected roughly 14 years after Wave I. The fourth wave included 15,700 respondents between the ages of 24 and 34. Add Health participants provided written informed consent for participation in all aspects of Add Health in accordance with the University of North Carolina School of Public Health Institutional Review Board guidelines. The University of North Carolina Institutional Review Board has approved the research reported as part of R01HD061622. Information about obtaining access to Add Health data can be found at http://www.cpc.unc.edu/projects/addhealth.

The analytic sample consists of respondents present in all four waves (N = 10,120). An additional seven respondents were dropped who were missing data for sex, race, or educational attainment (N = 10,113). Multiple imputation via chained equations was used to address missing data in the remaining covariates by constructing 10 complete data sets. The number of complete data sets, 10, was chosen to help ensure stable parameter estimates and standard errors [40]. Most of the covariates had missing data for less than 5 percent of respondents, but parental income was missing for a greater percentage of respondents (22 percent). Diagnostics were performed on the imputed values for parental income and results indicated that the imputed values did not significantly alter the distribution of the measure.

### Measures

The measures of perceived attractiveness come from two items completed by interviewers at each wave (we use Waves 1, 2, and 3). The first item is “how physically attractive is the respondent?” and the second item is “how attractive is the respondent’s personality?” For both items interviewers rated respondents from 1 “very unattractive” to 5 “very attractive.” For both dimensions of attractiveness, ratings averaged between 3 (“about average”) and 4 (“attractive”) at every wave (see Table 1).

**Table 1:** Descriptive statistics (N = 10,113).

	Mean		Mean
Educational attainment	3.15	Parental education	2.99
		Parental income (logged)	3.53
*Physical attractiveness*		*Personality attractiveness*	
Wave 1	3.57	Wave 1	3.60
Wave 2	3.58	Wave 2	3.61
Wave 3	3.50	Wave 3	3.64
*Other covariates*			
Age (wave 1)	15.30	Two biological parents	.55
Female	.55	Two parents	.17
White	.55	Single mother	.20
Black	.21	Single father	.03
Hispanic	.15	Other family structure	.05
Other race	.09	Peabody vocabulary test	100.81
West	.25	Self-reported GPA (wave 1)	2.79
Midwest	.26	Body mass index (wave 1)	22.56
South	.37		
Northeast	.12		

*Notes*: Means are averages over the 10 complete data sets. Educational attainment and parental education range from 1 “less than high school education” to 5 “more than a four-year college degree.” All attractiveness measures range from 1 “very unattractive” to 5 “very attractive.”

The outcome for this analysis is educational attainment measured at Wave IV. Values range from 1 “less than high school education” to 5 “more than a four-year college degree.” The study relies on two measures of parent socioeconomic resources. The first is the highest level of education completed by either parent, coded the same as respondent education. The second is logged family income reported by the parent. Parent education and parent income are both obtained from a parent questionnaire. Respondents had an average attainment between some postsecondary education and four-year degree while parental education averaged close to some postsecondary education (see Table 1). Both respondent education and parental education are treated as continuous measures in the following analyses.

The models include a number of potential confounders that may be related to parent socioeconomic resources and influence both perceived attractiveness and educational attainment. The confounders include sociodemographic measures: the age of respondents at Wave IV, sex, race (non-Hispanic white, non-Hispanic black, Hispanic, and other race), family structure in adolescence (two-biological parents, two parents, single mother, single father, and other family structure), and the region of the United States in which the respondent lived in adolescence (west, midwest, south, and northeast). Three additional potential confounders capture cognitive/academic ability and body size: Peabody picture vocabulary test [41], self reported GPA at Wave I when respondents were in high school, and adolescent BMI.

### Statistical Models

The analytic approach relies on extracting the stable components (“traits”) of perceived physical and personality attractiveness by specifying each dimension of attractiveness as a latent variable measured by the three indicators over time. The latent variables for attractiveness are then incorporated into a larger model of predictors of educational attainment. The larger model allows for the specification of interactions between the latent variables for attractiveness and the measures of parent socioeconomic resources. The model without interactions can be represented by the following system of equations

$$edu_{i}=\alpha +\beta_{1}{\left(L\;phy\right)}_{i}+\beta_{2}{\left(L\;per\right)}_{i}+\beta_{3}pedu_{i}+\beta_{4}pinc_{i}+\beta_{5}^{'}x_{i}+\epsilon_{i}$$ (1)

$$phy_{ij}=\alpha_{phy,j}+\lambda_{phy,j}{\left(L\;phy\right)}_{i}+\delta_{phy,j}$$ (2)

$$per_{ij}=\alpha_{per,j}+\lambda_{per,j}{\left(L\;per\right)}_{i}+\delta_{per,j}\;,$$ (3)

where i indexes respondents, j indexes waves 1, 2, and 3, *edu* is respondent’s education, *L phy* and *L per* are latent perceived physical and personality attractiveness respectively, *phy* and *per* are the measures of perceived physical and personality attractiveness across the three waves, *pedu* and *pinc* are parental education and logged parental income, and **x** is a vector of additional covariates. Interactions between latent attractiveness measures and parent socioeconomic resources can be included in the first equation.

The analysis proceeds in three steps. The first step is to develop a measurement model for perceived physical attractiveness and attractive personality. The second step is to specify structural equation models that treat the two latent attractiveness variables as mediators in the educational attainment model. The third step is to test whether parental education and parental income moderate any effects of attractiveness on educational attainment. Parameters estimated based on the models in the second and third steps allow us to assess our primary research questions: (1) whether associations between physical/personality attractiveness and educational attainment vary by parental socioeconomic resources and (2) whether parental socioeconomic resources are associated with perceived physical and personality attractiveness.

Auxiliary analyses were conducted to analyze whether the relationships observed in the overall sample hold for subgroups defined by sex and race, whether the observed relationships held net of specific personality dimensions, and whether the observed relationships held in a sibling subsample. Past studies suggest that standards of attractiveness are more salient for women than men and that there are also racial and ethnic differences in the assessment of attractiveness [42]. Despite these differences, however, empirical studies have generally not found that perceived attractiveness has a greater effect for different demographic subgroups [5,13]. Nonetheless, the first auxiliary analysis examines whether the role of perceived attractiveness in mediating and/or moderating the link between parent socioeconomic resources and educational attainment differs by sex and race/ethnicity.

The second auxiliary analysis adds measures of the Big Five personality traits [43] to the models to help determine whether specific dimensions of personality account for any potential relationship between personality attractiveness and educational attainment. Wave IV of Add Health includes the Mini-IPIP, a 20-item short-form version of the International Personality Item Pool that is designed to measure the Big Five factors of personality [44]. These items were used to construct scales for agreeableness, conscientiousness, extraversion, imagination, and neuroticism.

The third auxiliary analysis tests whether associations between perceived attractiveness and educational attainment remain after accounting for unobserved family factors that may shape both attractiveness and educational attainment. This analysis leverages the sibling subsample of Add Health to specify sibling fixed effects models to address confounding by unobserved family factors. The sibling subsample consists of 2,766 siblings from 1,383 families with nonmissing data for educational attainment. Missing data in the covariates were handled via multiple imputation with chained equations by creating 10 complete data sets. The sibling fixed effects model was specified to incorporate latent measures of perceived physical and personality attractiveness in addition to covariates that vary within sibling pairs (Peabody vocabulary test scores, grade point average, and body mass index) [45].

The analyses involve specifying structural equation models in which latent variables (perceived physical and personality attractiveness) interact with observed variables (parental education and log parental income). The numerical procedures for estimating a model involving latent interactions do not permit the inclusion of sample weights [46]. Because of this, the analyses do not use the Add Health sample weights to adjust for unequal probabilities of inclusion and attrition over time. Instead, the analyses rely on including the primary variables that account for the unequal probabilities of inclusion in the sample in the models [47].

The data were prepared for analysis using Stata v13 [48] and the structural equation models were estimated using Mplus v7 [46]. All of the Stata and Mplus code for the analysis is available for replication and further analysis at a github repository (https://github.com/sbauldry/attr).

## Results

### Measurement Model for Perceived Attractiveness

The first step in the analysis is to develop a measurement model for the two types of perceived attractiveness. A natural starting point is a model that specifies a latent variable for perceived physical attractiveness and a latent variable for perceived personality attractiveness each measured by their respective indicators across the first three waves of data (Model 1). Interviewers might have a tendency to rate respondents high or low on both physical attractiveness and attractive personality at any given wave independent of the trait component of each measure. To capture this possibility, a second model specification allowed the disturbances for physical attractiveness and attractive personality to be correlated at each wave (Model 2).

Table 2 presents model fit statistics and selected parameter estimates for Models 1 and 2. The overall model fit statistics strongly support the model allowing for correlated disturbances over the baseline model. Although the chi-square statistic remains significant in Model 2, all of the other measures of model fit suggest that the model is a good fit with the data—the BIC is negative, the CFI and TLI are both close to 1, and the RMSEA is below .05. In addition, the correlation between latent physical and personality attractiveness is estimated to be 1.31, an impossible value, in Model 1, which is another indication of poor model fit.

**Table 2:** Measurement Model Fit Statistics and Selected Parameter Estimates (N = 10,113).

	Model 1		Model 2	
	Physical	Personality	Physical	Personality
*Factor Loadings*				
Wave 1 indicator	1	1	1	1
	-	-	-	-
Wave 2 indicator	1.02	1.06	1.16	1.15
	[.95, 1.09]	[.98, 1.14]	[1.02, 1.30]	[.99, 1.31]
Wave 3 indicator	.60	.64	.59	.54
	[.54, .66]	[.58, .70]	[.53, .65]	[.46, .62]
Cor(Phy, Per)	1.31		.72	
	[1.27, 1.35]		[.69, .75]	
*Reliabilities*				
Wave 1 indicator	.28	.23	.25	.23
Wave 2 indicator	.34	.28	.39	.32
Wave 3 indicator	.11	.09	.10	.06
*Model Fit Statistics*				
Chi-square	6757.38		17.21	
df	8		5	
BIC	6683.61		-28.90	
CFI	.52		1.00	
TLI	.11		1.00	
RMSEA	.29		.02	

*Notes*: Model fit statistics and estimates averaged over 10 complete data sets. The factor loadings are unstandardized estimates with 95% confidence intervals in brackets. Model 1 is the baseline model. Model 2 allows the disturbances between the indicators for a given wave to be correlated. The factor loadings for the Wave 1 indicators for both attractive personality and physical attractiveness are fixed to 1 to identify the model.

In Model 2, the Wave 2 and Wave 3 indicators for perceived physical attractiveness and attractive personality are strongly associated with their respective latent variables as indicated by the factor loadings (the factor loadings for the Wave 1 indicators are fixed to 1 in order to identify the model). The correlation between the two dimensions of perceived attractiveness (r = .72) is positive and moderate-to-high in magnitude. Finally, the estimated reliabilities reveal that the indicators of attractiveness contain a notable amount of measurement error. The reliabilities at best come close to .4, but are below .1 for the Wave 3 indicators. These low reliabilities underscore the importance of extracting the trait components of perceived attractiveness from the noisy measures. Thus, the analysis proceeds with the second measurement model to introduce latent perceived attractiveness into the educational attainment model.

### Perceived Attractiveness and Educational Attainment

Steps 2 and 3 of the analysis incorporate the measurement model for perceived physical and personality attractiveness into a model for educational attainment and introduce interactions between the latent dimensions of perceived attractiveness and parent socioeconomic resources. Table 3 presents selected parameter estimates from the model incorporating the two dimensions of perceived attractiveness and from a set of four additional models that add interactions between the two dimensions of perceived attractiveness and the two measures of parent socioeconomic resources (the complete set of parameter estimates is available at the github repository referenced above).

**Table 3:** Selected Parameter Estimates from Educational Attainment Models (N = 10,113).

	Educational Attainment						
	Model 1	Model 2	Model 3	Model 4	Model 5	Per	Phy
Parent education	0.18	0.28	0.20	0.18	0.18	0.02	0.02
	[.16, .20]	[.21, .35]	[.17, .23]	[.16, .20]	[.16, .20]	[.01, .03]	[.01, .03]
Parent income (logged)	0.13	0.13	0.13	0.13	0.12	0.03	0.06
	[.10, .16]	[.10, .16]	[.10, .16]	[.02, .24]	[.08, .16]	[.01, .05]	[.04, .08]
Per attractiveness	0.30	0.53	0.30	0.31	0.30		
	[.18, .42]	[.33, .73]	[.18, .42]	[.01, .61]	[.18, .42]		
Phy attractiveness	0.06	0.06	0.25	0.06	0.00		
	[-.04, .17]	[-.05, .16]	[.08, .42]	[-.04, .17]	[-.28, .28]		
Per X par edu		-0.07					
		[-.12, -.02]					
Phy X par edu			-0.06				
			[-.10, -.02]				
Per X par inc				0.00			
				[-.07, .07]			
Phy X par inc					0.02		
					[-.05, .09]		
R-square	0.41	-	-	-	-	0.25	0.22

*Notes*: Unstandardized estimates with 95% confidence intervals in brackets. R-squares are not available for the models including an interaction with a latent variable. Par edu refers to parent education, par inc refers to parent income (logged), phy refers to latent physical attractiveness, and per refers to latent personality attractiveness.

The first column of Table 3, labeled M1, reports selected estimates for the predictors of educational attainment. As expected, both parental education and logged parental income have positive associations with educational attainment (b = .18 and b = .13 respectively). Perceived personality attractiveness also has a positive association with educational attainment (b = .30), but perceived physical attractiveness does not. The size of the personality effect on educational attainment, when standardized, is comparable to the effect of parental income, about half the effect of parental education, and about a third of the effect of GPA.

Associations between other covariates (not shown in Table 3) and educational attainment are largely consistent with past studies. Women, people who grew up with both biological parents (as compared with other two parent households and single mother households), people living in the Northeast (as compared with people living in the West), and people with more facility with English and cognitive abilities (as indicated by Peabody vocabulary test scores and GPA) are all associated with higher levels of education. In addition, people living in the South have slightly lower levels of education than people living in the West.

**Fig 1:**
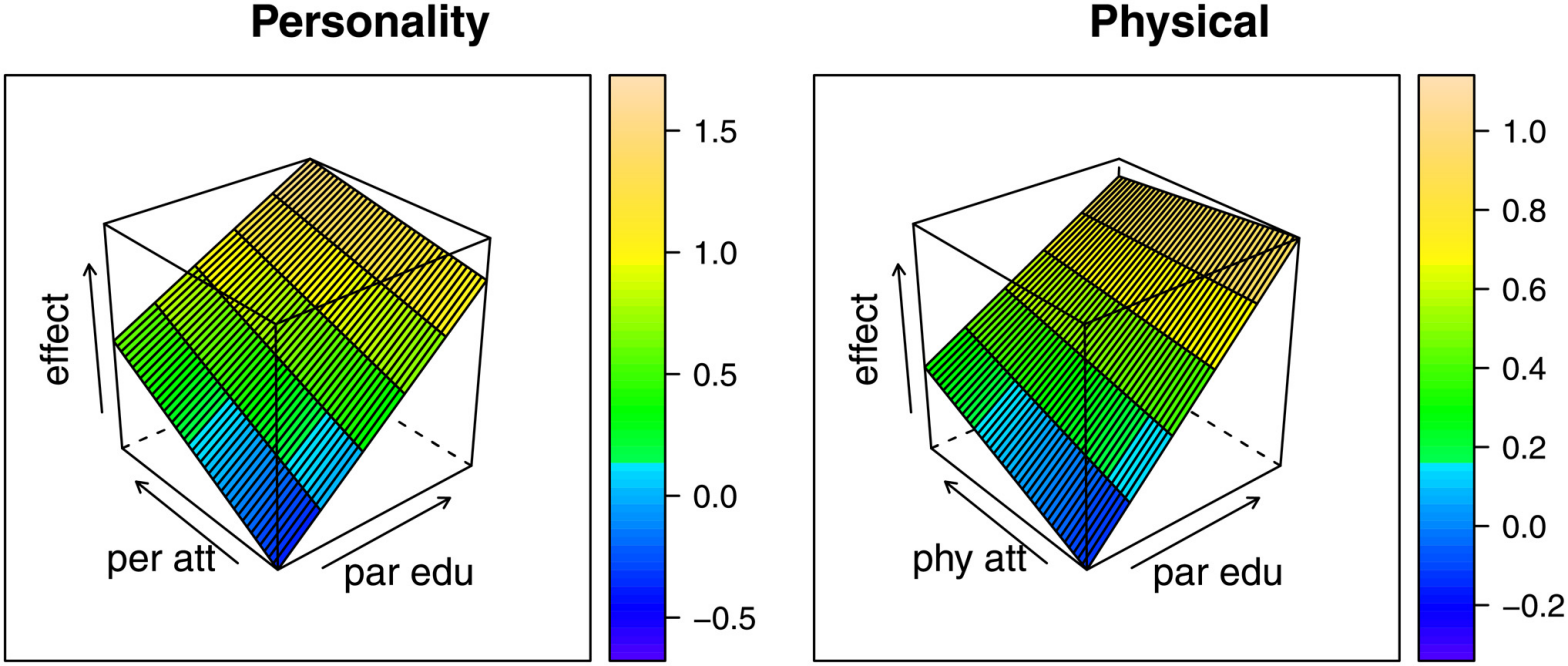
Response surface plot illustrating interaction of latent perceived attractiveness and parental education on educational attainment. Parental education ranges from 1 to 5. The latent dimensions of perceived attractiveness have mean 0 and standard deviations .40 and .44 for personality attractiveness and physical attractiveness respectively. The graph thus captures roughly plus/minus 2 standard deviations. The z-axis illustrates how the effect on educational attainment varies across the ranges of perceived attractiveness and parental education based on the parameter estimates reported in Table 3, Models 2 and 3.

Models 2 through 5 introduce the interactions between parent socioeconomic resources and the two dimensions of perceived attractiveness. In the model that includes an interaction term between parental education and perceived personality attractiveness (M2), the two main effects (parental education b = .13, personality attractiveness b = .53) and the interaction effect (b = -.07) are all predictors of educational attainment. This pattern of positive main effects and a negative interaction indicates that the association between perceived personality attractiveness and education is reduced at higher levels of parental education (see Fig 1, Panel A). This is apparent in the steeper slope for personality attractiveness at lower levels of parental education than at higher levels of parental education. In other words, as predicted, perceived personality attractiveness has a stronger association with educational attainment at lower levels of parental education.

Model 3 includes an interaction term between parental education and perceived physical attractiveness. In this model, a similar pattern of estimates is observed as found with perceived personality attractiveness: the two main effects are positive (parental education b = .13, physical attractiveness b = .25) and the interaction effect is negative (b = -.06). Once again, this particular pattern of effects suggests that the association between perceived physical attractiveness and educational attainment is stronger at lower levels of parental education (see Fig 1, Panel B).

Models 4 and 5 introduce interactions between parental income and both dimensions of perceived attractiveness. In both models the confidence intervals for the interaction terms overlap 0 and thus there is no evidence that the associations between perceived attractiveness and educational attainment vary across different levels of parental income.

The last two columns of Table 3 report estimates of the associations of parent socioeconomic resources with latent perceived personality and physical attractiveness respectively. Parental education has positive associations with both perceived personality attractiveness (b = .02) and perceived physical attractiveness (b = .02). Similarly, logged parental income also has positive associations with both perceived personality attractiveness (b = .03) and perceived physical attractiveness (b = .06). The sizes of the effects are not large, but, when standardized, are comparable in magnitude to the effect of BMI on perceived personality attractiveness and to about half of the effect of being female on perceived physical attractiveness.

Interestingly, other positive correlates of each dimension of perceived attractiveness include women, Hispanics (as compared with whites), and people with more facility with English and cognitive abilities (as indicated by Peabody vocabulary test scores and GPA). People with higher BMIs in adolescence are perceived as less physically attractive and as having a less attractive personality.

### Auxiliary Analyses: Sex and Race/Ethnicity

Auxiliary analyses by sex and race/ethnicity were conducted to determine whether the patterns observed in the full sample held among different subgroups. Parameters for the two measurement models for perceived attractiveness outlined above were estimated separately for females and males and for whites, blacks, Hispanics, and other racial/ethnic groups. For all subgroups the second model fit the data better than the first, and the parameter estimates for the second model (i.e., the factor loadings, correlation between the two latent dimensions of attractiveness, and the reliabilities of the indicators) were similar to the overall measurement model. Therefore, the second measurement model specification that allowed correlated disturbances between the indicators of physical and personality attractiveness at each given wave was used for all subgroups.

Separate models by sex revealed a similar pattern of results as observed in the full sample (results available at the github repository referenced above). Parental education, parental income, and perceived personality attractiveness have associations with educational attainment for both females and males. The interactions between parental education and perceived personality attractiveness and between parental income and perceived personality attractiveness exhibit the same pattern of positive main effects and negative interaction effect, but they are only statistically significant among females. Finally, parental education and parental income have positive associations with both dimensions of perceived attractiveness for females and males; however, the confidence interval for the association between parental education and perceived physical attractiveness among females includes 0.

Separate models by race/ethnicity also reveal a similar pattern of results as observed in the full sample, though with fewer statistically significant associations (results available at the github repository referenced above). Parental education, parental income, and perceived personality attractiveness all have positive associations with educational attainment among every racial/ethnic group with the one exception of parental income among Hispanics. In the models including interactions between parent socioeconomic resources and perceived attractiveness, the only interaction is between perceived physical attractiveness and parental education among Hispanics. For all racial/ethnic groups, however, the pattern of positive main effects for parent socioeconomic resources and perceived personality attractiveness and a negative interaction remains. Furthermore, the estimates for the associations between parental socioeconomic resources and perceived attractiveness are all similar in magnitude to the estimates in the full sample, though some are not statistically significant.

These results indicate that the role of perceived attractiveness in the link between parent socioeconomic resources and educational attainment operates similarly among females and males and among whites, blacks, Hispanic, and other races. The reduced statistical power from considering members of each group separately renders some of the associations non-significant, but there is no evidence that perceived attractiveness plays a different role in the educational attainment process by sex or race/ethnicity.

### Auxiliary Analysis: Big Five Dimensions of Personality

The second auxiliary analysis examined whether including the Big Five dimensions of personality (agreeableness, conscientiousness, extraversion, imagination, and neuroticism) in the model attenuated the association between personality attractiveness and educational attainment and/or altered the pattern of resource substitution. Adjusting for the specific dimensions of personality attenuates the effect of personality attractiveness on educational attainment by 27 percent (b = .30 in primary analysis and b = .22 in auxiliary analysis), but an association between personality attractiveness and educational attainment remains. In addition, the inclusion of the Big Five dimensions of personality does not alter the pattern of resource substitution described above.

### Auxiliary Analysis: Sibling Fixed Effects

The third auxiliary analysis relies on sibling fixed effects models to test whether associations between perceived attractiveness and educational attainment remain once one accounts for family-level unobserved confounders. In fact, both latent personality attractiveness (b = .32) and latent perceived attractiveness (b = .41) maintain positive associations with educational attainment net of Peabody vocabulary test scores, grade point average, and body mass index at wave 1. Thus the relationship between perceived attractiveness and educational attainment does not appear to be substantially affected by unobserved family level confounders.

## Discussion

Detailed understanding of the intergenerational reproduction of status and mobility is of critical importance given recent trends in American society that document growing inequality. How do the children of less well-educated parents “get ahead?” Evidence shows that achievement is not solely based on relevant merits. Yet the extent to which other forms of capital contribute to status is not well understood. The purpose of this study was to examine (1) whether the association between attractiveness and educational attainment varies by parent socioeconomic resources and (2) whether parent socioeconomic resources are associated with attractiveness.

With respect to the first research question, the results suggest that both perceived personality and physical attractiveness have stronger associations with educational attainment for people coming from families with low levels of parental education. In other words, perceived attractiveness may compensate for other background disadvantages with respect to educational attainment, but people from disadvantaged backgrounds are less likely to be perceived as attractive. This pattern of results extends the resource substitution with structural amplification theory developed by Ross and Mirowsky beyond health outcomes to the status attainment process and non-cognitive traits.

The results also indicate that perceived personality attractiveness has a positive association with educational attainment. This finding suggests that past studies that have focused exclusively on physical attractiveness appear to be missing the more important dimension of personality attractiveness, which reflects factors other than the Big Five personality dimensions. Given the excellent content validity of the Big Five, the issue for future research becomes what, beyond the Big Five, makes people’s personality attractive? Plausible candidates include interpersonal warmth, body language (e.g., eye contact), cooperativeness, and motivational characteristics. Yet plausible candidates must contend with the fact that our measure of “personality attractiveness” reflected an impression that the respondent made on the interviewer during a short visit.

Additionally, most past studies have not addressed measurement error in the indicators of attractiveness, and thus past findings of physical attractiveness may be biased. Given the notable magnitude of measurement error that we observe, reliance on state-based measures may be highly misleading. It is often asserted that measurement error leads to attenuation of effects, but this is only true in bivariate analyses. In analyses involving numerous covariates, particularly covariates that may also contain non-negligible measurement error, the bias can be in any direction [49].

With respect to the second research question, the results indicate that parent socioeconomic resources have a small association with both dimensions of perceived attractiveness. Nonetheless, the magnitudes of the associations are roughly comparable with the associations between BMI and perceived personality attractiveness and about half of the gap between females and males in perceived physical attractiveness.

Why do these forms of attractiveness matter for attainment? Prior research suggests that attractive students may elicit attention from teachers and coaches and unrelated adult mentors [34]. Yet the mechanisms are not known and deserve further attention, particularly to inform interventions to assist educators.

### Limitations

One important limitation of this study is that estimates should not be interpreted as causal effects. Although the study is able to incorporate a wide array of potential confounders, including two measures of ability and adolescent body size, and the relationships between perceived attractiveness and educational attainment remain once one adjusts for unobserved family-level confounders, additional unmeasured within-family confounders could influence parent socioeconomic resources, perceived attractiveness, and educational attainment.

An additional limitation of this study is that there is only a single rating of attractiveness for each respondent within a given wave. Ideally, the data would have included multiple interviewers’ ratings of the physical and personality attractiveness of each respondent at each wave. Such information would have allowed for an assessment of the within-wave reliability of the attractiveness measures. Lacking these data, analyses cannot correct for the potential systematic bias in some interviewers’ assessments of attractiveness at each wave. This limitation is one reason, however, why we chose to focus on attractiveness across waves as the longitudinally-defined trait helps to counter systematically over- or under-rated attractiveness at any given wave, especially given that interviewers differed across waves.

## Conclusions

Drawing on nationally representative data, trait-based measures of attractiveness, and an engaging conceptual model, our paper suggests that, in contrast to students from higher class households, attractiveness promotes educational attainment moreso among students from lower class backgrounds, but that these same students are less likely to be judged attractive. This moderating pattern reflects “resource substitution,” and there is now mounting evidence that diverse forms of capital—social, psychological, physical, financial—may be interchangeable.

The potential interchangeability of different forms of capital opens important avenues for future work on the reproduction of inequality. Precisely how these forms of capital “substitute” for one and other and translate into “value” in the classroom is not well understood and should be the subject of further investigation. Such knowledge would not only improve our understanding of the contours of social stratification but could also have practical implications in helping teachers foster learning in students from diverse backgrounds and with diverse constellations of capital.
